# Feasibility of predicting next-day fatigue levels using heart rate variability and activity-sleep metrics in people with post-COVID fatigue

**DOI:** 10.3389/fdgth.2025.1689846

**Published:** 2025-11-18

**Authors:** Nana Yaw Aboagye, Maria Germann, Kenneth F. Baker, Mark R. Baker, Silvia Del Din

**Affiliations:** 1 Translational and Clinical Research Institute, Faculty of Medical Sciences, Newcastle University, Newcastle upon Tyne, United Kingdom; 2 NIHR Newcastle Biomedical Research Centre, Newcastle upon Tyne, United Kingdom; 3 Newcastle Upon Tyne Hospitals NHS Foundation Trust, Newcastle upon Tyne, United Kingdom; 4 Department of Clinical Neurophysiology, Royal Victoria Infirmary, Newcastle upon Tyne, United Kingdom

**Keywords:** post-COVID syndrome, fatigue prediction, machine learning, heart rate variability, accelerometry, wearable technology, XGBoost, digital biomarkers

## Abstract

**Background:**

Post-COVID fatigue (pCF) represents a significant burden for many individuals following SARS-CoV-2 infection. The unpredictable nature of fatigue fluctuations impairs daily functioning and quality of life, creating challenges for effective symptom management.

**Objective:**

This study investigated the feasibility of developing predictive models to forecast next-day fatigue levels in individuals with pCF, utilizing objective physiological and behavioral features derived from wearable device data.

**Methods:**

We analyzed data from 68 participants with pCF who wore an Axivity AX6 device on their non-dominant wrist and a VitalPatch electrocardiogram (ECG) sensor on their chest for up to 21 days while completing fatigue questionnaires every other day. HRV features were extracted from the VitalPatch single-lead ECG signal using the NeuroKit Python package, while activity and sleep features were derived from the Axivity wrist-worn device using the GGIR package. Using a 5-fold cross-validation approach, we trained and evaluated the performances of two machine learning models to predict next-day fatigue levels using Visual Analogue Scale (VAS) fatigue scores: Random Forest and XGBoost.

**Results:**

Using five-fold cross-validation, XGBoost outperformed Random Forest in predicting next-day fatigue levels (mean R² = 0.79 ± 0.04 vs. 0.69 ± 0.02; MAE = 3.18 ± 0.63 vs. 6.14 ± 0.96). Predicted and observed fatigue scores were strongly correlated for both models (XGBoost: r = 0.89 ± 0.02; Random Forest: r = 0.86 ± 0.01). Key predictors included heart rate variability features—sample entropy, low-frequency power, and approximate entropy—along with demographic (age, sex) and activity-related (moderate and vigorous duration) factors. These findings underscore the importance of integrating physiological, demographic, and activity data for accurate fatigue prediction.

**Conclusions:**

This study demonstrates the feasibility of combining heart rate variability with activity and sleep features to predict next-day fatigue levels in individuals with pCF. Integrating physiological and behavioral data show promising predictive accuracy and provides insights that could inform future personalized fatigue management strategies.

## 1 Introduction

Post-COVID fatigue (pCF) represents a significant and persistent symptom affecting 10%–35% of individuals following SARS-CoV-2 infection, even amongst those who only experienced mild initial symptoms of COVID-19 (1, 2). This debilitating symptom can persist for months or even years, severely impacting daily functioning, employment status, and overall quality of life (3). The World Health Organization has recognized post-COVID condition (PCC) as a major health concern, with fatigue consistently reported as one of the most prevalent and disabling symptoms (4). An estimated 1.3 million people in the UK are experiencing self-reported long COVID, with fatigue being the most reported symptom. This accounts for approximately 51% of individuals affected by long COVID (5).

The management of pCF presents unique challenges due to its unpredictable nature and complex etiology, which likely involves dysregulation of multiple physiological systems including autonomic nervous system dysfunction and circadian rhythm disruption (6, 7). Traditional fatigue management approaches often rely on retrospective symptom reporting via fatigue questionnaires and general energy conservation strategies, which are subjective and fail to account for the day-to-day variability in fatigue levels (8). This unpredictability of fatigue creates significant barriers to activity planning, pacing strategies, and effective symptom management, contributing to reduced quality of life, impaired work capacity, and increased healthcare utilization (9, 10).

The ability to predict next-day fatigue levels would represent a significant advancement in pCF management, enabling patients to proactively adjust their activity levels and implement targeted interventions before fatigue exacerbations occur. Recent advances in wearable technology and artificial intelligence have opened new possibilities for continuously monitoring physiological and behavioral parameters that may predict symptom fluctuations (11, 12). Wearable devices can capture detailed patterns of physical activity, rest, and sleep while providing physiological measurements such as heart rate variability (HRV) that might serve as digital biomarkers for fatigue prediction (13, 14).

HRV, reflecting autonomic nervous system function, has emerged as a promising physiological biomarker for fatigue and stress-related conditions. HRV features derived from time-domain (RMSSD, pNN50, SDNN), frequency-domain (LF/HF ratio, HF power), and non-linear analysis (SD1, SD2, entropy measures) provide insights into autonomic regulation that may be disrupted in post-COVID conditions. Integrating HRV with activity and sleep patterns offers a more comprehensive understanding of fatigue mechanisms and prediction. However, the complex, non-linear relationships between these physiological and behavioral parameters and next-day fatigue, particularly in pCF, remain poorly understood.

Several studies have explored the relationship between activity patterns and fatigue in chronic conditions such as multiple sclerosis (15), cancer-related fatigue (16), and myalgic encephalomyelitis/chronic fatigue syndrome (ME/CFS) (17). These investigations have identified potential associations between fatigue levels and various activity metrics, including daily step count (18), and time spent in some activity intensity levels like moderate-to-vigorous physical activity (MVPA) (19). Sleep parameters such as sleep efficiency, duration, and timing have also been linked to daytime fatigue in various populations (20, 21).

Traditional statistical approaches often struggle to capture these complex relationships due to their reliance on linear associations and predefined interaction terms (22). Machine learning (ML) techniques, on the other hand, can identify complex patterns in high-dimensional data without requiring *a priori* assumptions about feature relationships (23). These methods have shown promise in predicting symptom fluctuations in various chronic conditions (24, 25). Some studies have demonstrated the utility of ML approaches for classifying fatigue levels in immune and neurodegenerative disorders using gait variability, with support vector machines and other classification methods showing promise for fatigue identification (11). However, their application to pCF fatigue prediction using combined physiological and behavioral data remains limited. Tree-based ensemble methods, like Random Forest and XGBoost, are particularly suited for this task, as they can capture non-linear feature interactions while providing interpretable feature importance rankings (26).

This study aims to address these gaps by exploring machine learning models for next-day fatigue level prediction in individuals with pCF using a combination of heart rate variability and activity/sleep metrics. Specifically, we employ two distinct machine learning frameworks:

1. Random Forest: An ensemble learning method that builds multiple decision trees and averages their predictions, particularly effective at capturing complex feature interactions while providing robust feature importance estimates (27).
2. XGBoost: An optimized gradient boosting implementation that provides superior performance through iterative refinement of predictions, with built-in regularization to prevent overfitting (28).

By combining physiological HRV features with behavioral activity and sleep patterns, we aim to predict the next day's fatigue state using previous-day data while gaining insights into the relative importance of different physiological and behavioral factors. This approach addresses a critical need in pCF research: developing tools to predict fatigue fluctuations using objective, continuously monitored parameters. By identifying the key predictors of next-day fatigue, this work could inform strategies for behavioral and therapeutic interventions, though we acknowledge that association does not imply causation, and interventional studies would be needed to establish causal relationships.

## 2 Methods

### 2.1 Study design and participants

#### 2.1.1 Study design and ethical approval

This investigation was conducted as part of a larger single-site, single-blind, sham-controlled, randomized research study examining the efficacy of non-invasive vagus nerve stimulation on post-COVID fatigue syndrome (https://research.ncl.ac.uk/covidfatigue/). The protocol received full ethical approval from the Faculty of Medical Sciences Research Ethics Committee at Newcastle University (Medical School, Framlington Place, NE2 4HH, UK) [Reference: (2284/18447/2021)] and the Declaration of Helsinki was followed in all study procedures, and the trial was registered in the ISRCTN registry, trial number (ISRCTN18015802).

#### 2.1.2 Participant Recruitment and Eligibility Criteria

Recruitment was conducted between May 2022 and April 2024 through multiple channels, including the dedicated trial website (covidfatigue.co.uk) and various social media platforms. Potential participants were initially screened using an electronic implementation of the Fatigue Impact Scale. Participants were eligible for inclusion if they met the following criteria: (1) adults aged 18–65 years; (2) documented positive COVID-19 test result without requiring hospitalization; (3) minimum of 4 weeks post-diagnosis; (4) self-reported fatigue affecting daily functioning; (5) desire for fatigue treatment; and (6) English language fluency. Exclusion criteria comprised: (1) pre-existing neurological or psychiatric disorders; (2) cardiac disease (including cardiomyopathy, myocardial infarction, arrhythmia, or prolonged QT interval); (3) presence of implanted electronic devices (e.g., pacemakers); (4) pregnancy or breastfeeding; and (5) contraindications for transcranial magnetic stimulation.

#### 2.1.3 Sample characteristics

While the trial enrolled 114 participants, not all had complete datasets for VitalPatch physiological monitoring, activity monitoring, and questionnaires due to technical issues, including Bluetooth connectivity problems, device malfunctions, and varying compliance with questionnaire completion. Therefore, our analysis focused on 68 participants with complete and usable data across these domains. Demographic and clinical characteristics of the analytical sample are presented in Table 1.

**Table 1:** Sample characteristics.

Characteristic	Value
Participants	68
Total observations	383
Sex	
Male	19
Female	49
Age (years)	
Mean ± SD	47.9 ± 12.0
Range	0–64
Treatment condition	
nVNS	24
Sham	23
Placebo	21
VAS fatigue score	
Mean ± SD	63.1 ± 16.1
Range	19–100

#### 2.1.4 Intervention protocol

Participants were randomized to one of three intervention conditions, all utilizing transcutaneous electrical nerve stimulation (TENS) via a FlexiStim device applied to the external ear:

1. Active taVNS: Stimulation of the auricular branch of the vagus nerve via the tragus (nVNS wing)
2. Sham control: Resistor-modified clip on the tragus designed to prevent actual stimulation
3. Electrical stimulation control: Stimulation of the greater auricular nerve via the earlobe (Placebo)

The intervention protocol required participants to self-administer the assigned treatment three times daily for eight weeks. During the second phase of the trial, control group participants crossed over to active stimulation so that all participants received active stimulation during the study.

### 2.2 Data collection

#### 2.2.1 Activity, sleep assessment and electrocardiography

Participants wore two devices during the study period:

Wearable device for activity and sleep assessment: An Axivity AX6 device (Axivity Ltd, UK) was worn continuously on the participant's non-dominant wrist for up to 21 days (14 days at baseline, 7 days at follow-up). The AX6 is a small (23 × 32.5 × 8.9 mm), lightweight (11 g) triaxial accelerometer with built-in temperature and light sensors. The device was configured to record raw acceleration data at 100 Hz with a dynamic triaxial accelerometer range of ±8 g, and a triaxial gyroscope range of ±2,000 degrees per second. This was passive monitoring requiring no participant interaction with the device.

ECG measurement: A VitalPatch biosensor (VitalConnect, Campbell, CA, USA) was utilized to collect continuous ECG data from participants during baseline and follow-up periods, each lasting 7 days. The device was worn on the left chest and adhered to the skin using a fully disposable, medical-grade patch. It features a single-lead ECG sensor, a triaxial accelerometer, and a thermistor, recording ECG at a sampling frequency of 125 Hz. Equipped with a zinc-air battery, the device lasts up to 7 days and can store up to 10 h of data locally, transmitting encrypted recordings wirelessly to a secure cloud platform for remote monitoring. The ECG data collected by VitalPatch facilitated the extraction of heart rate variability (HRV) features. Participants were instructed to wear the device continuously.

#### 2.2.2 Fatigue assessment

Participants completed fatigue assessments every other day at any time during the day throughout the study using a Visual Analogue Scale (VAS) delivered through a secure web application. Each assessment day, participants rated their average fatigue level on a scale from 0 (no fatigue) to 100 (worst fatigue imaginable). The VAS for fatigue has demonstrated good reliability and validity in various clinical populations (29).

### 2.3 Data processing

#### 2.3.1 Activity, sleep and HRV data processing

Raw accelerometer data from the Axivity AX6 device were processed using the validated GGIR package (version 2.7-1) (30) in R (version 4.1.0) (31), which handled auto-calibration, non-wear detection, and the extraction of physical activity, sleep, and circadian rhythm features from 24-hour wrist-worn recordings. A summary of all Activity and sleep features and their definitions is provided in Table 2.

**Table 2:** Activity features.

Activity category	Feature type	Description
**Physical activity**	ACC_	Accelerometry-based activity measures
	dur_day_	Duration of daytime activity periods
	dur_night_	Duration of nighttime activity periods
	quantile_	Activity intensity quantiles
	Total physical activity	
**Sleep metrics**	sleep_	Sleep duration and quality metrics
	efficiency	Sleep efficiency measures
	Total sleep metrics	
**Circadian rhythm**	M5	Most active 5-hour period
	L5	Least active 5-hour period
	Total circadian rhythm	

ECG data from the VitalPatch sensors were processed to derive HRV features using the NeuroKit2 Python library. The HRV analysis pipeline consisted of: (1) ECG signal preprocessing and quality control, (2) R-peak detection and RR interval extraction, (3) segmentation into 5-minute windows, (4) computation of standard time-domain, frequency-domain, and non-linear HRV features for each window, and (5) aggregation of these features into daily summaries using 12 statistical descriptors (mean, median, standard deviation, minimum, maximum, first and third quartiles, interquartile range, range, coefficient of variation, skewness, and kurtosis). A summary of all HRV features and their definitions is provided in Table 3. Movement artifacts were addressed through multiple approaches: (1) chest-mounted device positioning to minimize motion artifacts,(2) automated quality control and artifact removal via NeuroKit2's built-in algorithms, (3) 5-minute windowing to ensure local stationarity of the signal, and (4) robust statistical aggregation (median, interquartile range, etc.) that naturally down-weights outlier windows affected by movement. HRV was computed across all 24-hour periods rather than isolated rest states to capture the full range of autonomic function throughout daily activities.

**Table 3:** HRV features.

HRV category	Feature	Description
**Time domain**	Mean_RR	Mean RR interval between consecutive heartbeats (ms)
	SDNN	Standard deviation of NN intervals, measuring overall HRV
	RMSSD	Root mean square of successive RR interval differences
	pNN50	Percentage of successive RR intervals differing by >50ms
	SDSD	Standard deviation of successive RR interval differences
	Total time domain	
**Frequency domain**	VLF_Power	Very low frequency power (0.003–0.04 Hz)
	LF_Power	Low frequency power (0.04–0.15 Hz)
	HF_Power	High frequency power (0.15–0.4 Hz)
	LF_HF_Ratio	Low frequency to high frequency power ratio
	Total frequency domain	
**Geometric**	SD1	Standard deviation perpendicular to line of identity (Poincaré plot)
	SD2	Standard deviation along line of identity (Poincaré plot)
	SD1_SD2_Ratio	Ratio of short-term to long-term variability
	Total geometric	
**Nonlinear**	ApEn	Approximate entropy, measuring regularity of heart rate patterns
	SampEn	Sample entropy, measuring pattern complexity in RR intervals
	DFA_alpha1	Short-term detrended fluctuation analysis scaling exponent
	DFA_alpha2	Long-term detrended fluctuation analysis scaling exponent
	Total nonlinear	

Overall, a comprehensive set of daily features was derived from the recordings and grouped into four primary domains:

1. Activity features (e.g., mean acceleration, intensity-specific time, activity bouts, and fragmentation indices),
2. Sleep features (e.g., total sleep duration, sleep efficiency, timing, and movement-based parameters),
3. Circadian rhythm features (e.g., M5, L5, and relative amplitude),
4. HRV features, reflecting autonomic nervous system function based on ECG-derived RR intervals.

In total, 279 daily features were extracted per participant per day: 192 from HRV and 82 from activity, sleep, and circadian rhythm domains, plus 5 demographic and treatment condition variables, age, sex, and intervention condition dummy variables). (Tables 2,3 show a breakdown of all the activity, sleep, circadian rhythm and HRV features used).

#### 2.3.2 Data preprocessing and feature engineering

Prior to model training, comprehensive data quality control was implemented. Exact duplicate observations were identified and removed from the dataset. Participants with less than 2 days of complete data were excluded to ensure adequate data representation. Missing values were handled through complete case analysis, removing any observations with missing feature values.

Treatment conditions were consolidated into three categories (nVNS, Sham, Placebo) and converted to dummy variables for model input. Sex was encoded numerically (Male = 0, Female = 1). All continuous features were standardized using z-score normalization to ensure equal contribution to model training.

To predict next-day fatigue, we aligned the accelerometer and HRV features from each day (day n) with the fatigue score reported on the following day (day n + 1). This temporal alignment ensures that models learn to predict future fatigue states based on current physiological and behavioral patterns, reflecting real-world application scenarios where predictions would be made for the following day.

### 2.4 Predictive modeling

#### 2.4.1 Model selection and implementation

We implemented two machine learning modeling approaches:

1. Random Forest (RF) is an ensemble of decision trees trained on bootstrap samples from the data. We utilized the “RandomForestRegressor” implementation in the scikit-learn package in Python, with hyperparameters optimized via grid search cross-validation.
2. XGBoost is a gradient boosting algorithm that builds trees sequentially, with each tree correcting errors made by earlier trees. We utilized the “XGBRegressor” package in Python, with learning rate, maximum tree depth, regularization parameters, and other hyperparameters optimized through extensive grid search.

Both models were optimized using a comprehensive grid search with 5-fold cross-validation to identify the best hyperparameter combinations for minimizing mean absolute error (MAE). The grid search explored multiple hyperparameter combinations for each model.

#### 2.4.2 Training and evaluation strategy

We employed a rigorous 5-fold cross-validation methodology to ensure robust assessment of model performance and prevent overfitting. While participant-level validation (leave-one-subject-out cross-validation) would provide the most stringent test of generalizability across individuals, this approach was not feasible given our data structure. With participants contributing varying amounts of data (range: 2–11 days, median: 3.0 days), many participants had insufficient observations to serve as meaningful test sets in a leave-one-subject-out framework.

Therefore, we implemented standard 5-fold cross-validation where the complete dataset was randomly divided into five equal-sized, non-overlapping segments (folds) using stratified sampling to maintain representative distributions of fatigue scores across folds. This approach led to an 80%/20% train/test division for each fold, where four segments (80% of the data, approximately 306 observations) were used for model training while the remaining segment (20%, approximately 77 observations) acted as the independent test set. While this approach may include data from the same participants in both training and test sets within a fold, it provides more stable performance estimates given our data constraints and reflects the practical scenario where prediction models would be personalized to individual users over time.

The cross-validation process was implemented as follows: For each of the five iterations, one-fold was held out as the test set while the remaining four folds formed the training set. Hyperparameter optimization was performed exclusively on the training data using nested cross-validation to prevent data leakage. Feature scaling (standardization) was fit on the training data only and then applied to the test data to ensure no information from test sets influenced model training. This process was repeated five times, with each segment serving as the test set exactly once, ensuring that every observation was used for testing once while being utilized for training four times. Final performance metrics were calculated as the mean and standard deviation across all five folds, providing robust estimates of model generalizability.

For comprehensive model evaluation, we quantified performance using the following metrics:

1. R2 score: Proportion of variance in fatigue levels explained by the model.
2. Mean Absolute Error (MAE): Average absolute prediction error between predicted and actual fatigue levels.
3. Correlation coefficient: A Measure of linear association between predicted and actual fatigue levels.

#### 2.4.3 Feature importance analysis

For tree-based models (RF and XGBoost), we used their built-in feature importance methods, which measure how much each feature contributes to prediction accuracy. For Random Forest, this indicates the mean decrease in impurity (Gini importance). In contrast, for XGBoost, it indicates the gain in information for each feature based on its contribution to model predictions.

We calculated feature importances from each fold of the 5-fold cross-validation process to ensure robust feature importance estimates and then aggregated these across all folds. This approach provides more stable rankings of importance and accounts for variability across different training sets. For each model and each fold, we extracted the feature importances attribute and then calculated:

1. Mean importance: Average importance across all five folds
2. Standard deviation: Variability in importance across folds
3. Consistency score: A Measure of how consistently important a feature is across folds

This cross-validation-based feature importance analysis guarantees that our rankings accurately reflect the learned relationships during model evaluation, rather than relying on importance calculated from a single training set. Features were ranked by their mean importance across all folds.

## 3 Results

### 3.1 Participant characteristics

The final analysis dataset included 389 observations from 68 unique participants with a mean age of 47.3 ± 11.5 years (range: 0–64 years). Participants were distributed across intervention conditions as follows: nVNS (*n* = 24 participants), Sham (*n* = 23 participatns), and placebo (*n* = 21 participatns).

Data availability varied across participants, with an average of 3.5 days of data per participant (range: 1–11 days, median: 3.0 days). This variation in data availability reflects the every-other-day assessment schedule and individual differences in questionnaire completion rates. All participants had complete data for the features included in the final models, and the processed dataset used for machine learning analysis had no missing values.

### 3.2 Model performance

**Figure 1:**
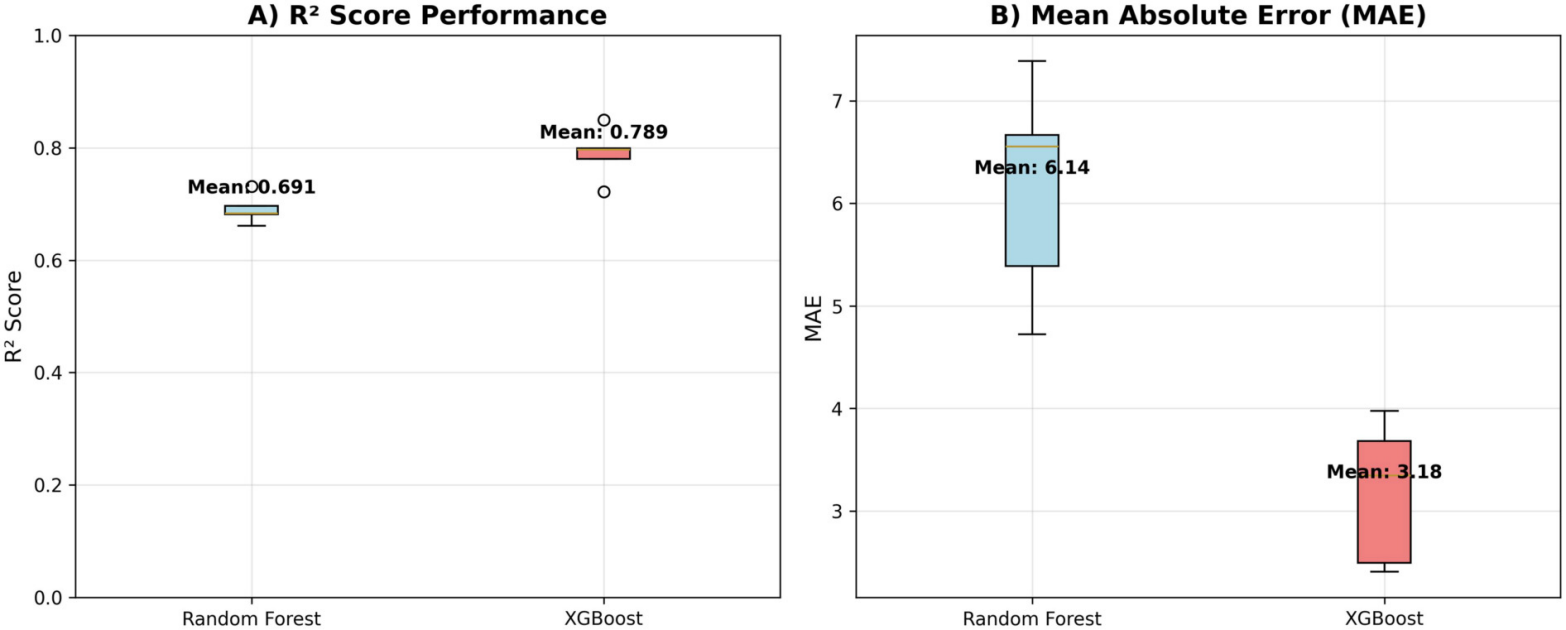
Model performance metrics. R² and MAE scores for both machine learning models. **(A)** R² values. **(B)** MAE values.

**Figure 2:**
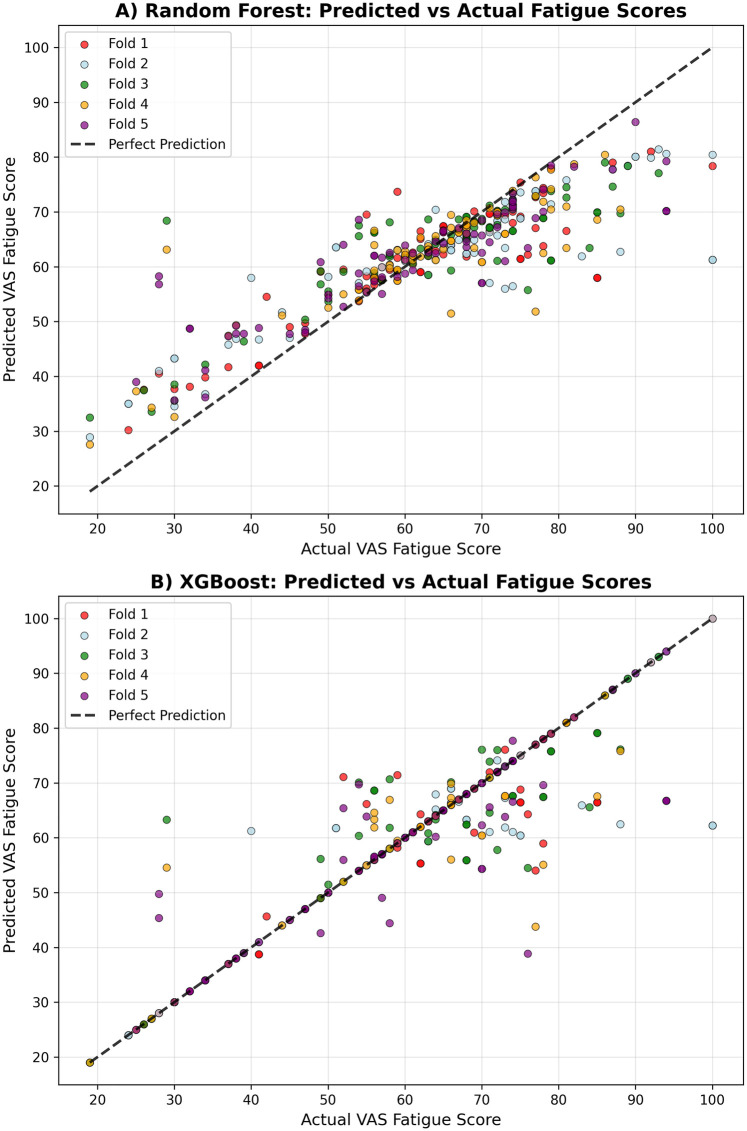
Prediction accuracy comparison. Correlation between observed and predicted fatigue scores for each model. **(A)** Random Forest. **(B)** XGBoost.

Table 4 presents the cross-validated performance metrics for each model across all five folds. The XGBoost model demonstrated the strongest predictive performance, achieving a mean R² score of 0.789 ± 0.041 and a mean absolute error of 3.18 ± 0.63 (see Figure 1). The model showed excellent correlation between predicted and actual fatigue scores (r = 0.892 ± 0.022) (see Figure 2). Random Forest achieved a mean R² score of 0.691 ± 0.023 with a mean absolute error of 6.14 ± 0.96 and correlation coefficient of 0.864 ± 0.013.

**Table 4:** Model results.

Model	Metric	Mean	SD	Fold_1	Fold_2	Fold_3	Fold_4	Fold_5
Random forest	R² Score	0.691	0.023	0.731	0.681	0.661	0.697	0.683
Random forest	MAE	6.14	0.96	5.39	7.39	6.67	4.72	6.56
Random forest	Correlation	0.864	0.013	0.878	0.876	0.845	0.852	0.872
XGBoost	R² Score	0.789	0.041	0.849	0.799	0.796	0.78	0.722
XGBoost	MAE	3.18	0.63	2.49	3.34	3.68	2.41	3.98
XGBoost	Correlation	0.892	0.022	0.925	0.899	0.893	0.886	0.856

Both tree-based models demonstrated strong predictive capability, with XGBoost showing superior performance across all metrics. The consistently high correlation coefficients (>0.86 for both models) indicate strong linear relationships between predicted and actual fatigue scores, while the R² values demonstrate that both models can explain a substantial proportion of variance in next-day fatigue levels. The cross-validation standard deviations indicate good stability of performance across different data splits, with Random Forest showing slightly more consistent performance (lower standard deviation) than XGBoost.

**XGBoost Top 5 Features:**

LF_Power_min—Low frequency power minimum

SDSD_std—SDSD standard deviation

ApEn_mean—Approximate entropy mean

SDSD_kurtosis—SDSD kurtosis

ApEn_q1—Approximate entropy Q1

**Random Forest Top 5 Features:**

SampEn_cv (- Sample entropy coefficient of variation

SampEn_kurtosis—Sample entropy kurtosis

LF_Power_cv—Low frequency power coefficient of variation

age_at_baseline—Age

SDNN_kurtosis—SDNN kurtosis

The feature importance analysis revealed several key patterns:

HRV Dominance: Heart rate variability features, particularly non-linear measures (sample entropy variability, approximate entropy) and frequency domain characteristics (low-frequency power), emerged as the most important predictive features. These metrics reflect autonomic nervous system function and heart rate complexity.

Demographic Factors: Age emerged as a significant predictor in the Random Forest model (ranked 4th), while sex appeared in the top 10 for Random Forest, suggesting potential demographic differences in fatigue patterns or responses to physiological and behavioral factors.

**Figure 3:**
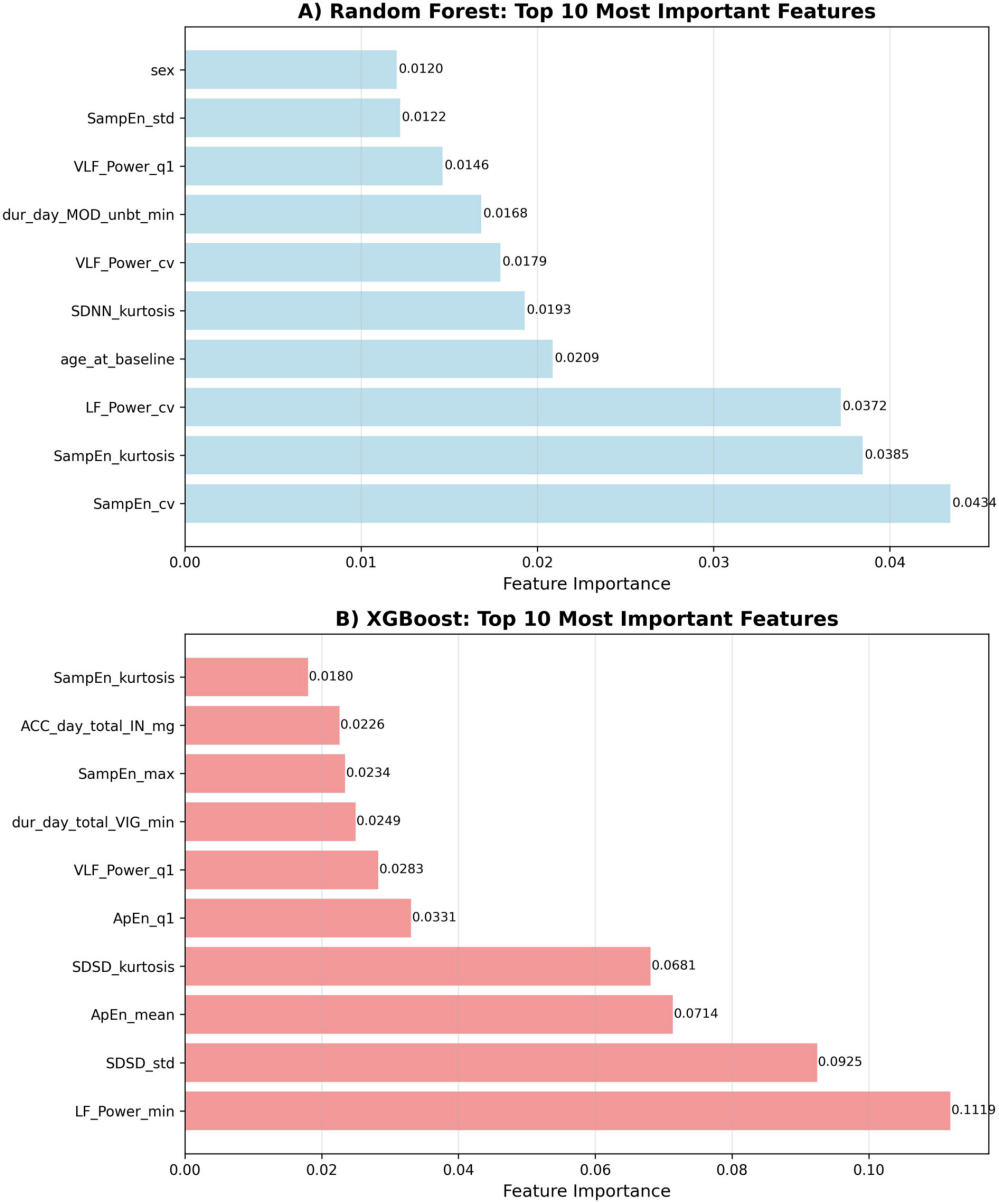
Feature importance rankings. Top 10 most influential predictors of next-day fatigue identified by each model. **(A)** Random Forest. **(B)** XGBoost.

Activity Patterns: Features related to the distribution and timing of physical activity were essential, particularly duration of moderate and vigorous activity periods, supporting the relationship between activity levels and next-day fatigue (see Figure 3).

## 4 Discussion

This pilot study has demonstrated the feasibility of using machine learning approaches to predict next-day fatigue levels in individuals with post-COVID fatigue based on objective physiological and behavioral metrics from wearable devices. Our findings suggest that it is possible to achieve a moderate prediction accuracy for next day fatigue state in participants with Long Covid by combining heart rate variability measures with activity and sleep patterns, indicating the potential for digital biomarkers to inform personalized fatigue management strategies in future larger-scale studies.

### 4.1 Key findings

#### 4.1.1 Intervention control and model robustness

Intervention condition (nVNS, Sham, Placebo) was included as dummy variables in all predictive models to control for potential treatment effects. The final analysis included 68 unique participants (19 males, 49 females) with 389 total observations distributed across intervention groups: nVNS (*n* = 24 participants, 35.3%), Placebo (*n* = 23 participants, 33.8%), and Sham (*n* = 21 participants, 30.9%). Intervention control analysis revealed that model performance remained robust when intervention variables were excluded from the feature set. For XGBoost, R² decreased only slightly from 0.789 ± 0.041 (with intervention) to 0.759 ± 0.043 (without intervention), while Random Forest showed minimal change from 0.691 ± 0.023 to 0.689 ± 0.018. This indicates that the physiological and behavioral patterns captured by our features predict next-day fatigue independent of intervention status, demonstrating the generalizability of our predictive models across different treatment conditions.

#### 4.1.2 Model performance

The performance of the tree-based models, particularly XGBoost, demonstrates that next-day fatigue prediction involves complex, non-linear interactions between physiological and behavioral features. XGBoost outperformed Random Forest likely due to its advanced gradient boosting algorithm and built-in regularization capabilities, which better handle feature interactions and prevent overfitting in this complex prediction task. This finding aligns with previous research in symptom prediction for chronic conditions, showing that ensemble methods often outperform traditional statistical approaches (33). The high correlation coefficients (>0.88) and significant variance explained (R2 score >0.8) indicate that objective wearable-derived metrics provide some form of predictive power for next-day fatigue levels. The achieved mean absolute errors of 3.04 and 6.54 for XGBoost and Random Forest, respectively, represent clinically meaningful prediction accuracy on the 0–100 VAS fatigue scale, as these fall within ranges that could inform practical decision-making for fatigue management. While minimal clinically important differences (MCID) for VAS fatigue scales in post-COVID populations have not been fully established, our prediction errors are minimal given a scale of a 0–100.

#### 4.1.3 Feature importance

The emergence of HRV metrics as the strongest predictors represents a significant finding, highlighting the role of autonomic nervous system dysfunction in post-COVID fatigue. Frequency domain measures (LF_power), and non-linear measures (SampEn) consistently ranked among the top predictors, suggesting that autonomic dysregulation may be a key mechanism underlying fatigue fluctuations in pCF. This finding aligns with previous research demonstrating that real-world cardiorespiratory measures can stratify participants with persistent fatigue in other conditions, supporting the utility of physiological monitoring for fatigue assessment (34).

The importance of activity patterns, particularly moderate-to-vigorous physical activity duration and moderate activity bouts aligns with the energy envelope theory and the concept of post-exertional malaise observed in ME/CFS (35). Stratified analyses revealed that morning activity patterns (M5TIME < 12 h) were associated with superior predictive performance (XGBoost R² = 0.792 ± 0.106) compared to afternoon (R² = 0.070 ± 0.985) and evening (R² = 0.525 ± 0.438) patterns. These findings suggest that the distribution and timing of activity throughout the day, rather than simply the total amount, play a crucial role in determining next-day fatigue levels, with morning activity patterns showing higher predictability.

While some sleep-related variables (sleep duration, sleep efficiency) were included in the model, they did not rank among the most important predictors, suggesting that physiological measures (HRV) and activity patterns are more predictive of next-day fatigue than sleep metrics in this population.

Demographic factors, particularly sex and age, emerged as important predictors, suggesting potential gender and age-related differences in fatigue mechanisms or responses to physiological and behavioral factors. Stratified analyses revealed that models performed better for females (XGBoost R² = 0.703 ± 0.074, Random Forest R² = 0.694 ± 0.037) compared to males (XGBoost R² = 0.387 ± 0.321, Random Forest R² = 0.480 ± 0.206), with the difference reflecting both sample size disparities (49 vs. 19 unique participants) and distinct physiological patterns. This finding warrants further investigation into sex-specific approaches to fatigue management.

#### 4.1.4 Physiological vs. behavioral contributions

The dominance of HRV features (40%–50% of top predictors) suggests that physiological monitoring provides the strongest signals for fatigue prediction, while behavioral factors (activity patterns, sleep) contribute approximately 45%–50% of predictive information. This finding supports the integration of both physiological and behavioral monitoring for comprehensive fatigue assessment and management.

### 4.2 Clinical implications

#### 4.2.1 Personalized fatigue management

The strong predictive power of HRV metrics suggests that continuous autonomic monitoring could provide early warning signals for fatigue exacerbations. By identifying optimal HRV patterns associated with lower next-day fatigue, clinicians could develop personalized autonomic regulation strategies through interventions such as heart rate variability biofeedback, breathing exercises, or stress management techniques.

The importance of activity timing patterns indicates that personalized activity scheduling based on individual circadian rhythms and activity tolerance could help optimize next-day fatigue levels. Rather than generic activity recommendations, interventions could focus on identifying individual-specific timing windows and activity distributions associated with better autonomic function and lower fatigue.

Integrating physiological (HRV) and behavioral (activity/sleep) monitoring enables a more comprehensive approach to fatigue management than either domain alone. This multi-modal approach could inform personalized recommendations that address both autonomic regulation and behavioral pacing strategies.

#### 4.2.2 Treatment planning

The identification of HRV metrics—particularly SampEn and LF_power—as the strongest predictors indicates that interventions targeting autonomic nervous system regulation may be especially effective for post-COVID fatigue. Potential strategies include heart rate variability biofeedback training to enhance autonomic control, breathing exercises and mindfulness practices to promote parasympathetic activation, and activity pacing approaches that integrate HRV monitoring for real-time feedback and self-regulation.

The observed importance of activity timing patterns (M5TIME) highlights the potential role of circadian rhythm–based interventions, such as light therapy or chronotherapy, in optimizing activity distribution and reducing next-day fatigue. Morning activity patterns were associated with superior predictive performance (XGBoost R² = 0.792 ± 0.106) compared to afternoon (R² = 0.070 ± 0.985) and evening (R² = 0.525 ± 0.438) patterns, suggesting that promoting earlier daily activity may represent a viable behavioral strategy for fatigue management.

The marked gender differences in model performance (females: R² = 0.703 ± 0.074; males: R² = 0.387 ± 0.321) further suggest the need for personalized, sex-specific intervention frameworks. Tailoring protocols to reflect the distinct physiological and behavioral fatigue patterns observed in each sex could enhance the efficacy of treatment strategies.

Importantly, the robust performance of our models even after excluding intervention variables (XGBoost R² decreased only from 0.789 to 0.759) indicates that physiological and behavioral patterns can predict fatigue independently of treatment status, supporting the potential generalizability of these findings across different clinical and lifestyle contexts.

Nevertheless, these associations are correlational and should not be interpreted as causal. Further validation through randomized controlled trials is essential to determine whether interventions targeting these identified predictors can directly improve fatigue outcomes.

#### 4.2.3 Monitoring and prevention

The model's performance suggests that wearable-based prediction algorithms could serve as early warning systems for high-fatigue days. Real-time HRV monitoring combined with activity tracking could provide personalized alerts when physiological or behavioral patterns indicate an increased risk of next-day fatigue exacerbation.

Such systems could enable proactive intervention by alerting individuals when HRV patterns suggest increased fatigue risk, recommending activity modifications based on current physiological state, providing feedback on optimal timing for activities based on individual patterns, and guiding pacing decisions through integration of autonomic and activity data.

The dominance of physiological markers suggests that objective monitoring may be more reliable than subjective symptom reporting for fatigue prediction, offering a path toward more precise and personalized management approaches.

### 4.3 Limitations and future directions

#### 4.3.1 Study limitations

Several limitations should be considered when interpreting our findings. First, the sample size, while adequate for initial modeling, could be expanded for more robust validation, particularly given the heterogeneity of post-COVID conditions. Second, the monitoring period (14 days at baseline plus 7 days at follow-up) might not capture longer-term patterns in activity and fatigue that could be relevant for prediction. Seasonal variations and major life events could influence both predictors and outcomes over longer timeframes.

Third, our cross-validation approach used standard 5-fold CV rather than participant-level (leave-one-subject-out) validation due to the limited number of observations per participant. While this provides stable performance estimates, it may overestimate model performance compared to true cross-participant generalization, as data from the same participants can appear in both training and test sets within folds. The varying data availability across participants (2–11 days per participant) necessitated this methodological choice, but future studies with more extensive longitudinal data per participant would benefit from participant-level validation to better assess cross-individual generalizability.

Our study population was limited to those with access to wearable technology and the ability to complete digital assessments every other day, potentially limiting generalizability to all individuals with pCF. Additionally, while our cohort showed a higher proportion of women compared to men, this gender distribution aligns with some published long-COVID literature showing consistent female predominance (ranging from 56%–79% across major cohorts) (2, 32) and likely reflects genuine sex differences in post-viral fatigue susceptibility rather than sampling bias. However, single-site recruitment and limited ethnic diversity may limit generalizability to other geographic regions and populations with different demographic characteristics. The study was conducted in a single center with limited ethnic diversity, which may not reflect the broader post-COVID population globally. Furthermore, while we controlled treatment conditions, the study was conducted in the context of an intervention trial, which might have influenced participants’ activity patterns and fatigue reporting. Finally, these conclusions are based on a single cohort, and validation in external, independent cohorts would strengthen the generalizability of our findings.

#### 4.3.2 Future research

Future research should focus on validating these findings in larger, more diverse populations of individuals with pCF. Multicenter studies with extended monitoring periods (ideally 6–12 weeks per participant) would enhance generalizability and allow for examination of longer-term patterns. Such studies should aim to collect sufficient longitudinal data per participant to enable participant-level cross-validation, providing more stringent tests of cross-individual generalizability. Additionally, prospective validation studies could assess the real-world utility of predictive models for fatigue management.

Investigation of longer-term patterns and their impact on fatigue prediction could provide insights into adaptation processes and disease trajectories in pCF. Development of real-time monitoring systems based on these models represents a promising direction for clinical application. Such systems could provide personalized activity recommendations and early warnings of fatigue exacerbations, potentially improving self-management and quality of life.

Finally, investigation of causal relationships between identified predictors and fatigue through interventional studies would strengthen the evidence base for targeted interventions. Experimental manipulation of key predictors, such as activity distribution or sleep timing, could test causal hypotheses and inform intervention development.

## 5 Conclusions

This study demonstrates the feasibility of using machine learning approaches to predict next-day fatigue levels in individuals with post-COVID fatigue using objective physiological and behavioral metrics derived from wearable devices. The XGBoost model achieved strong performance (mean R² score = 0.789 ± 0.041, MAE = 3.18 ± 0.63), suggesting that combined HRV and activity/sleep metrics provide valuable predictive information for fatigue forecasting in larger studies.

Key findings include that heart rate variability features emerged as the strongest predictors of next-day fatigue levels, particularly non-linear measures such as sample entropy variability and approximate entropy, highlighting the importance of autonomic nervous system complexity monitoring. Activity timing patterns and demographic factors (age, sex) also showed significant predictive power. Finally, the integration of physiological (HRV) and behavioral (activity/sleep) data provides superior prediction compared to either domain alone.

These findings have important implications for personalized fatigue management strategies and the development of early warning systems for high-fatigue days. Future research should focus on validating these findings in larger, more diverse populations and developing real-time monitoring systems that integrate HRV and activity data. Such efforts could ultimately lead to more effective, personalized interventions for individuals living with post-COVID fatigue.

## Data Availability

The raw data supporting the conclusions of this article will be made available by the authors, without undue reservation.
